# TyG index and MAFLD severity are associated with subclinical LV dysfunction in T2DM patients: a cross−sectional study

**DOI:** 10.3389/fendo.2025.1749989

**Published:** 2026-01-27

**Authors:** Xiangsui Hu, Hanwei Chen, Ziying Wang, Yun Deng, Long Huang, Chunquan Zhang, Liangyun Guo, Shengbo Liu, Lingmin Liao

**Affiliations:** 1 Department of Ultrasound, The Second Affiliated Hospital, Jiangxi Medical College, Nanchang University, Nanchang, China; 2 Department of Ultrasound, The Second Affiliated Hospital of Xi’an Jiaotong University, Xi’an, China; 3 Department of Ultrasound, Gao’an People’s Hospital, Gao’an, Jiangxi, China; 4 Department of Oncology, The Second Affiliated Hospital, Jiangxi Medical College, Nanchang University, Nanchang, China; 5 Ultrasound Application Specialist, GE Healthcare, Nanchang, China

**Keywords:** FIB-4, global longitudinal strain, MAFLD, TyG index, type 2 diabetes

## Abstract

**Background:**

Metabolic dysfunction-associated fatty liver disease (MAFLD) is highly prevalent in type 2 diabetes mellitus (T2DM) and may contribute to early myocardial dysfunction. The Fibrosis-4 (Fib-4) index is a reliable marker of hepatic fibrosis, and the triglyceride-glucose (TyG) index is an effective indicator of insulin resistance (IR). Both are linked to various cardiovascular diseases (CVDs).However, their combined impact on subclinical left ventricular dysfunction (SLVD) remains unclear. This study aimed to explore the associations between hepatic fibrosis, IR, and SLVD in T2DM patients.

**Methods:**

We enrolled 270 T2DM patients between September 2024 and April 2025.MAFLD was diagnosed by ultrasonography, hepatic fibrosis was assessed using the Fib-4 index, and insulin resistance was estimated using the TyG index formula. Left Ventricular Global Longitudinal Strain (LV GLS) was measured by speckle-tracking echocardiography, with SLVD defined as absolute value of LV GLS <18%. T2DM patients were divided into three groups based on the presence of MAFLD and Fib-4 <1.3 or ≥1.3: non-MAFLD, MAFLD+Fib-4<1.3, and MAFLD+Fib-4≥1.3, and further stratified by the TyG median. Multivariable logistic regression models were used to evaluate the independent and interactive associations of Fib-4 index and TyG index with SLVD.

**Results:**

Compared to non-MAFLD patients, T2DM patients with MAFLD were younger, had a shorter duration of diabetes, and exhibited worsened lipid profile, with higher TyG values and lower LV GLS. MAFLD independently predicted SLVD (adjusted OR = 3.21, 95% CI: 1.64–6.29). Even in patients with Fib-4 <1.3, MAFLD was associated with higher SLVD risk (OR = 2.94), while advanced fibrosis further increased SLVD risk (OR = 3.68). TyG independently predicted SLVD (adjusted OR = 2.73, 95% CI: 1.48–5.03). Importantly, patients with both high Fib-4 (≥1.3) and high TyG (≥9.03) had the greatest SLVD risk (OR = 7.58), whereas advanced fibrosis alone was not significant.

**Conclusions:**

Fib-4 index and TyG index are independent predictors of SLVD in T2DM, and their coexistence exerts a synergistic effect. Combined assessment provides a simple, non-invasive tool for early risk stratification, highlighting the clinical importance of the liver–heart axis in identifying SLVD.

## 1 Introduction

Type 2 diabetes mellitus (T2DM) is a global chronic metabolic disorder characterized by chronic hyperglycemia and insulin resistance (IR), and it remains a major contributor to cardiovascular disease (CVD), which the leading cause of morbidity and mortality in this population (1, 2). Emerging clinical and experimental evidence indicates that metabolic dysfunction-associated fatty liver disease (MAFLD), which is highly prevalent in T2DM, is strongly linked to the onset and progression of CVD (3, 4). In particular, hepatic fibrosis not only represents disease progression within MAFLD but also an independent predictor of adverse cardiovascular outcomes (5).

Among the available tools to assess the severity of MAFLD, the fibrosis-4 (Fib-4) index is widely utilized due to its simplicity, non-invasiveness, and robust predictive accuracy (6, 7). By integrating age, platelets (Plt) count, aspartate aminotransferase (AST), and alanine aminotransferase (ALT), the Fib-4 index provides an efficient assessment of hepatic fibrosis burden (7). Prior studies have demonstrated that patients with Fib-4 values ≥1.3 often exhibit moderate to advanced fibrosis, a pathological condition closely associated with elevated CVD, including arrhythmias, myocardial remodeling, and progression to heart failure with preserved ejection fraction (HFpEF) (8, 9).

IR, a central feature of T2DM, has also been shown to contribute substantially to CVD (10, 11). The triglyceride-glucose (TyG) index, a reliable surrogate marker of IR, has been widely adopted for the early identification of diabetes, metabolic syndrome, and CVD. Derived from fasting plasma glucose (FPG) and triglycerides (TG), the TyG index demonstrates high predictive accuracy (12, 13). Recent studies have reported strong associations between higher TyG index values and cardiovascular dysfunction. Specifically, elevated TyG levels have been linked to early myocardial injury and reduced Left Ventricular Global Longitudinal Strain (LV GLS), even in individuals with preserved left ventricular ejection fraction (LVEF) (12, 14).

Subclinical left ventricular dysfunction (SLVD) serves as an early marker of myocardial impairment, yet it is difficult to detect with conventional echocardiography (15). LV GLS has emerged as a highly sensitive echocardiographic parameter for evaluating LV systolic function. Importantly, SLVD, defined as impaired GLS despite preserved LVEF, represents an early stage of diabetic cardiomyopathy and is strongly predictive of future heart failure (15, 16). Taken together, these observations indicate that MAFLD-related hepatic fibrosis and IR may act as important cardiometabolic factors predisposing patients with T2DM to early LV systolic dysfunction.

However, although both Fib-4 and the TyG index have individually been associated with CVD outcomes, their relationships with SLVD in patients with T2DM have not been systematically evaluated, and it is unclear whether the coexistence of hepatic fibrotic burden and insulin resistance confers additional risk of early myocardial dysfunction beyond each factor alone. Therefore, in a cohort of patients with T2DM, we aimed to investigate the association of MAFLD and its severity, assessed by ultrasonography and the Fib-4 index, together with the TyG index as a surrogate of IR, with SLVD defined by impaired LV GLS. We hypothesized that the presence and greater severity of MAFLD, as well as higher TyG levels, would be independently associated with increased odds of SLVD (absolute LV GLS <18%) after adjustment for conventional cardiovascular risk factors.

## 2 Methods

### 2.1 Study design and participants

This was a single-center cross-sectional observational study conducted at the Second Affiliated Hospital of Nanchang University between September 2024 and April 2025. Consecutive patients with T2DM attending the Department of Endocrinology were screened for eligibility.

Inclusion criteria were: (1) Age 18–75 years;(2)T2DM diagnosis per ADA guidelines (17). Exclusion criteria included: (1) known cardiac disease (history of heart failure; coronary artery disease; atrial flutter or atrial fibrillation, left or right bundle branch block, implantation of a pacemaker or implantable cardioverter-defibrillator; and congenital heart disease; (2) LVEF<53%; (3) stroke/intracerebral hemorrhage history; (4) estimated glomerular filtration rate (eGFR) <60 mL/min/1.73 m²; (5) uncontrolled hypertension (SBP ≥180 mmHg or DBP ≥110 mmHg); (6) active malignancy or systemic infection; (7) secondary hepatic steatosis (alcohol >30g/day, viral hepatitis, hepatotoxic drugs); and (8) inadequate echocardiographic images or incomplete data.

**Figure 1:**
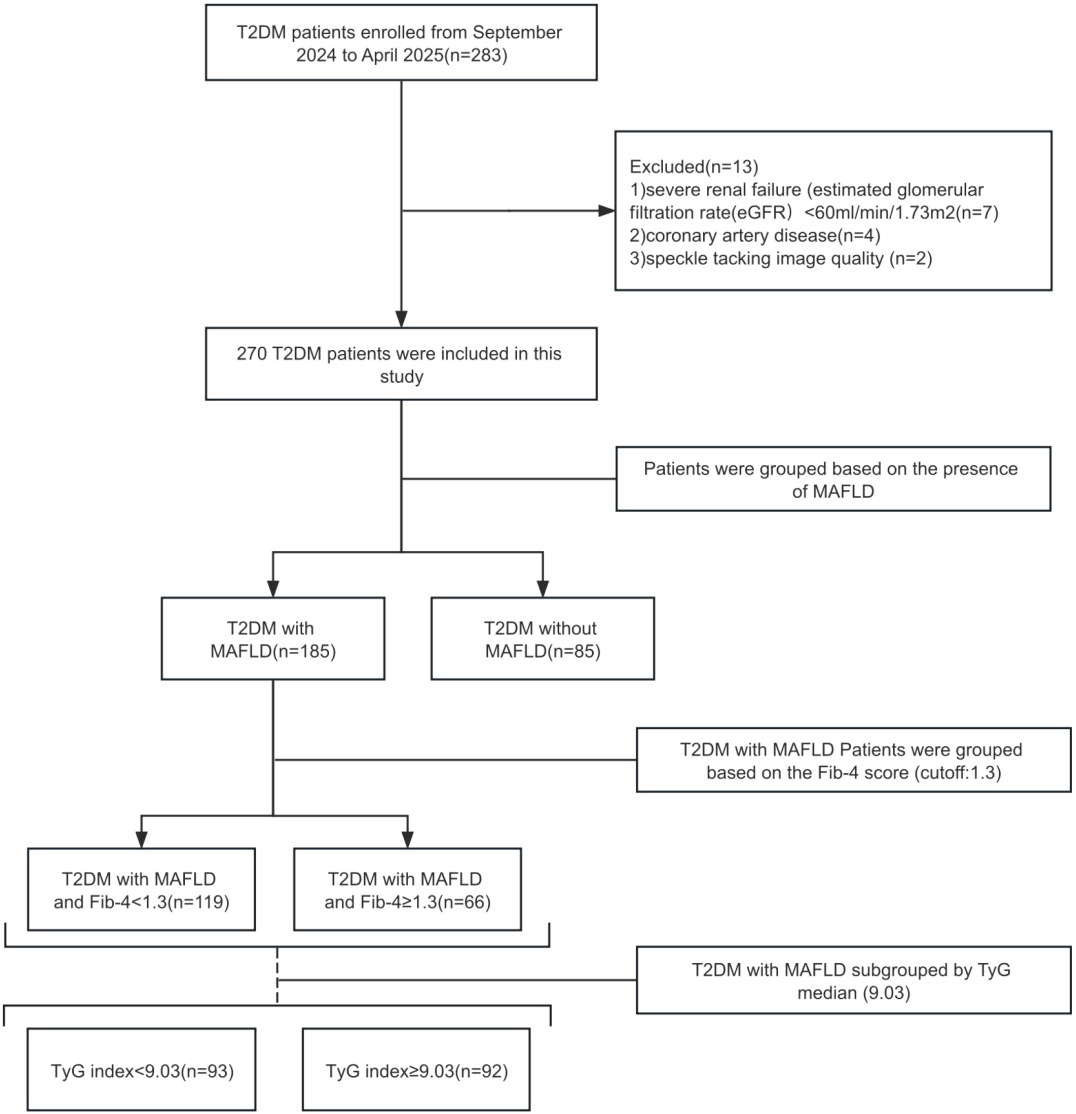
Study flowchart shows patient selection process.

Of the 283 screened T2DM patients, 13 were excluded according to the predefined criteria, leaving 270 patients for the final analysis (**Figure 1**). For descriptive analyses and primary comparisons, patients were first stratified into three groups according to the presence of MAFLD and the fibrosis-4 (Fib-4) index (cut-off 1.3): (1) patients without MAFLD; (2) patients with MAFLD and Fib-4 < 1.3; and (3) patients with MAFLD and Fib-4 ≥ 1.3. Among patients with MAFLD, we further stratified participants according to the median value of the TyG index to investigate the association between IR (as reflected by TyG) and SLVD in this population, and to explore whether this relationship differed across fibrosis strata.

The study protocol was approved by the Institutional Review Board of the Second Affiliated Hospital of Nanchang University (No. 2025-013) and was conducted in accordance with the Declaration of Helsinki. Written informed consent was obtained from all participants.

### 2.2 Clinical variables and biochemical measurements

Clinical data of were extracted from electronic medical records and included sex, age, body mass index (BMI), blood pressure, smoking status, diabetes duration (DM), and use of antidiabetic medications. Antidiabetic medications of interest included metformin, insulin and sodium glucose cotransporter-2 (SGLT2) inhibitors.

Laboratory assessments comprised glycated hemoglobin A1c(HbA1c), FPG, TC(Total Cholesterol), TG, high-density lipoprotein cholesterol (HDL-C), low-density lipoprotein cholesterol (LDL-C), Plt, ALT, AST, an d eGFR. Biochemical parameters are reported in SI units (e.g. mmol/L) according to our institutional laboratory. For the calculation of the TyG index, FPG and TG were converted to mg/dL using standard conversion factors, and the TyG index was computed as: TyG index = ln [TG (mg/dL) × FPG (mg/dL)/2 (13).

### 2.3 MAFLD diagnosis and fibrosis assessment

MAFLD was diagnosed according to international consensus criteria (18), based on ultrasonographic evidence of hepatic steatosis. All abdominal ultrasound examinations were conducted by experienced Ultrasound Physician blinded to the participants’ clinical and echocardiographic data. The diagnosis of hepatic steatosis was established according to standardized sonographic features, including increased echogenicity of the liver relative to the renal cortex, posterior beam attenuation, and impaired visualization of intrahepatic vascular structures and the diaphragm. Ultrasound has demonstrated good diagnostic accuracy for detecting mild to moderate hepatic steatosis. However, no semi-quantitative grading system was applied to assess the severity of steatosis in this study (19). Accordingly, steatosis was classified as present or absent.

To further evaluate hepatic fibrosis, the fibrosis-4 (Fib-4) index was calculated using the following formula:Fib-4 = [Age (years)× AST (IU/L)]/[Plt (×10^9^/L) × √ALT (IU/L)] (20). A cut-off of 1.3 was used to define indeterminate/advanced fibrosis, consistent with prior validation studies (20). This single cut-off was applied across the whole age range of our cohort, in line with previous MAFLD studies, although age-related differences in the diagnostic performance of Fib-4 have been reported.

### 2.4 Echocardiographic examination and definition of SLVD

Transthoracic echocardiography was performed by two experienced ultrasound physicians using a color Doppler ultrasound device equipped with an S5–1 phased-array probe (frequency range, 1.5–4.6 MHz) (GE Medical Systems, Milwaukee, WI, USA). Both physicians conducted the examinations independently in a double-blind manner, following to the guidelines set by the American Society of Echocardiography and the European Association of Cardiovascular Imaging (ASE/EACVI) (21). Conventional parameters included left atrial diameter, interventricular septum and posterior wall thickness of LV, LV end-diastolic and end-systolic volumes, and LVEF (Simpson’s biplane method) (21). All images were obtained in the left lateral decubitus position and recorded with ECG gating.

For speckle-tracking analysis, apical two-, three- and four-chamber views were acquired at a frame rate of 50–80 frames/s and stored as cine loops of at least three consecutive cardiac cycles.LV global longitudinal strain (GLS) was analyzed offline using vendor-specific software (EchoPAC, version 204, GE Medical Systems) by an experienced cardiologist who was blinded to all clinical and laboratory data. GLS values were originally exported as negative numbers; for ease of interpretation, all GLS values are reported as absolute values (%) in this manuscript. SLVD was defined as an absolute LV GLS <18% in the presence of preserved LVEF (21).

### 2.5 Statistical analysis

Continuous variables are expressed as mean ± standard deviation (SD) or median (interquartile range, IQR), as appropriate, and categorical variables as counts and percentages. Between-group differences were assessed using one-way analysis of variance (ANOVA) or the Kruskal–Wallis test for continuous variables and the chi-square test for categorical variables. When overall tests were statistically significant, pairwise comparisons were performed with appropriate *post-hoc* procedures.

Binary logistic regression was used to evaluate the associations between MAFLD status, Fib-4, the TyG index and SLVD. Three models were constructed: Model I, unadjusted; Model II, adjusted for age and sex; and Model III, fully adjusted for BMI, platelet count, diabetes duration, HbA1c, FPG, TC, HDL-C, ALT and antidiabetic medication use (metformin, insulin and SGLT-2 inhibitors). Odds ratios (ORs) and 95% confidence intervals (CIs) were reported.

To examine the joint association of hepatic fibrotic burden and IR with SLVD, patients were further stratified into four groups according to Fib-4 (<1.3 vs ≥1.3) and TyG (below vs above the median), using the low Fib-4/low TyG group as the reference category. This approach allowed us to assess whether the coexistence of elevated Fib-4 and TyG was associated with a higher likelihood of SLVD than either factor alone.

To address potential multicollinearity between the TyG index, its components (FPG, TC, TG) and other covariates, variance inflation factors (VIFs) were calculated in a linear regression model with GLS as the dependent variable and all predictors from Model III as independent variables, with VIF values >5 considered indicative of problematic multicollinearity.

In a randomly selected subset of 30 participants, intra- and inter-observer reproducibility of LV GLS measurements was assessed using intraclass correlation coefficients (ICC) with 95% CIs.

All statistical analyses were performed using SPSS version 26.0 (IBM Corp., Armonk, NY, USA). A two-sided P value <0.05 was considered statistically significant.

## 3 Results

### 3.1 Baseline characteristics of patients with type 2 diabetes stratified by MAFLD status and fibrosis severity

Among the 270 hospitalized T2DM patients included, 185 (68.5%) had MAFLD, while 85 (31.5%) were non-MAFLD. Of the 185 MAFLD patients, 66 (35.7%) exhibited intermediate/high fibrosis risk (FIib-4≥1.3), including 17 (9.2%) with high probability of advanced fibrosis (Fib-4≥2.67).

**Table 1** delineates baseline characteristics stratified by MAFLD status and fibrosis severity. Compared to non-MAFLD counterparts, MAFLD patients (irrespective of fibrosis) were younger with shorter DM, and exhibited adverse metabolic profiles including elevated TC, FPG, HbA1c, and lower HDL-C (all P <0.05). Sex distribution, blood pressure, hypertension prevalence, smoking history, and most liver enzymes (except AST) did not differ significantly across groups (P>0.05). Plt significantly across groups, with lower values in patients with advanced fibrosis (Fib-4 ≥ 1.3). The TyG index was significantly higher in both MAFLD groups compared to the non-MAFLD group (P < 0.001).Use of metformin and SGLT2 inhibitors was less frequent in the MAFLD group (P <0.01).

**Table 1:** Baseline clinical and metabolic characteristics of patients with type 2 diabetes stratified by MAFLD status and fibrosis severity.

Variables	Patients without MAFLD (n = 85)	Patients with MAFLD and Fib-4 < 1.3 (n = 119)	Patients with MAFLD and Fib-4 ≥ 1.3 (n = 66)	p value
Demographic Data				
Age (years)	57.3 ± 10.45	49.98 ± 11.36	60.1 ± 9.5	***<0.001***
Male sex (%)	64.7	68.1	62.1	0.69
Clinical Data				
BMI (kg/m^2^)	23.62 ± 4.09	25.93 ± 3.54	25.8 ± 2.94	0.12
Diabetes duration (years)	6(1,10)	3(1,8)	3(1,7)	***0.07***
Current smokers (%)	27.1	26.9	18.2	0.37
Systolic blood pressure (mmHg)	129.85 ± 19.33	129.63 ± 17.93	127.58 ± 12.24	0.32
Diastolic blood pressure (mmHg)	80.45 ± 13.02	84.89 ± 10.48	80.87 ± 11.75	0.25
Laboratory Data				
Plt (x 10^9^/L)	226.36 ± 92.02	252.86 ± 62.65	187.74 ± 44.28	***<0.001***
FBG (mmol/L)	7.8(6.02,11.15)	10.16(7.54,15.01)	8.95(7.22,12.34)	***<0.001***
HbA1c (%)	8.01 ± 2.36	9.73 ± 2.34	8.88 ± 2.39	***<0.001***
TG (mmol/L)	4.77 ± 1.3	5.18 ± 1.5	5.58 ± 2.59	0.24
LDL-C(mmol/L)	2.89 ± 0.98	3.24 ± 0.91	3.26 ± 1.14	0.10
HDL-C(mmol/L)	1.27 ± 0.3	1.11 ± 0.23	1.24 ± 0.25	***0.002***
TC(mmol/L)	1.32(0.8,1.97)	2.42(1.49,3.18)	1.70(1.37,2.72)	***<0.001***
AST (IU/L)	21.83(16.13,26.99)	19.85(16.17-24.21)	24.15(18.75-33.34)	***0.001***
ALT (IU/L)	19.6(14.49,28.05)	23.72(17.78,34.30)	22.23(16.39-30.62)	0.10
TyG Index	8.25 ± 0.81	9.00 ± 0.87	9.26 ± 0.89	***<0.001***
e-GFR (mL/min/1.73 m^2^)	99.15 ± 26.82	106.29 ± 24.29	101.88 ± 21.24	0.33
Medication Usage				
Insulin therapy (%)	22.4	18.5	19.7	0.79
Metformin (%)	55.3	31.1	21.2	***<0.001***
Sulphonylureas (%)	7.1	3.4	7.5	0.41
Pioglitazone (%)	8.2	7.6	4.5	0.69
Alpha-glucosidase inhibitors(%)	31.8	9.2	10.6	***<0.001***
DPP-4 inhibitors (%)	3.5	4.2	10.6	0.12
GLP-1 receptor agonists (%)	0	2.5	4.5	0.18
SGLT-2 inhibitors (%)	37.6	8.4	13.6	***<0.001***

Sample size, n = 270. Data are expressed as means± SD, medians and IQR (in parenthesis) or relative percentages. Differences between the three groups were tested by using the one-way analysis-of-variance (ANOVA) for normally distributed variables, the Kruskal-Wallis test for non-normally distributed variables (such as diabetes duration, serum triglycerides and liver enzymes) and the chi-squared test.

ALT, alanine aminotransferase; AST, aspartate aminotransferase; BMI, body mass index; DPP-4, dipeptidyl peptidase-4; e-GFR, estimated glomerular filtration rate; FPG, Fasting Plasma Glucose; Fib-4:Fibrosis-4 Index; HDL-C, High-Density Lipoprotein Cholesterol; HbA1c:Hemoglobin A1c; GLP-1, glucagon-like peptide-1; LDL-C, Low-Density Lipoprotein Cholesterol; MAFLD, metabolic dysfunction-associated fatty liver disease; Plt, Platelets; SGLT-2, sodium glucose cotransporter-2; TC, Total Cholesterol; TG, Triglycerides.

Bold values indicate statistical significance (P < 0.05).

**Figure 2:**
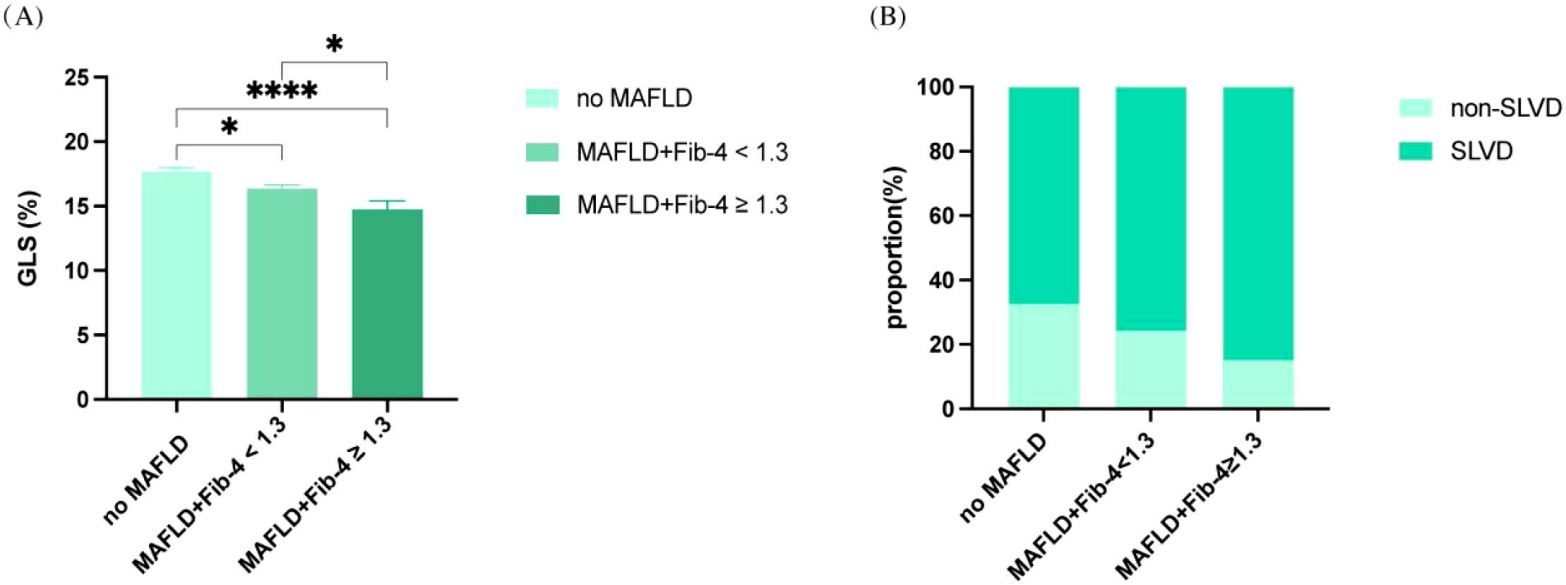
**(A)** Comparison of LV GLS across MAFLD severity groups: no MAFLD, MAFLD with Fib-4 < 1.3, and MAFLD with Fib-4 ≥ 1.3. Bar plot shows mean GLS (%) in patients with each group. GLS progressively declined with increasing MAFLD severity. Error bars represent the standard error of the mean (SEM). Group differences were assessed using one-way ANOVA with post-hoc pairwise comparisons. *P < 0.05, ****P < 0.0001 for post-hoc comparisons vs no MAFLD. **(B)** Prevalence of SLVD across the same MAFLD severity groups. Association between MAFLD severity and SLVD in T2DM patients.

No significant differences were observed between the groups in LA, IVS, LVPW, LVEDD, LVESD, LVMi, LVEF (P>0.05) (**Table 2**). However, there was a significant difference in the E/e’ ratio (P < 0.001), with the highest values observed in the MAFLD + Fib-4 ≥ 1.3 group. Additionally, LV GLS was significantly lower in the MAFLD groups, particularly in the MAFLD + Fib-4 ≥ 1.3 group, where the lowest GLS was observed (17.69 ± 2.97 vs. 16.35 ± 3.12 vs. 15.33 ± 3.19, overall P < 0.001) (**Figure 2A**). The prevalence of SLVD was markedly higher in patients with MAFLD, especially in those with elevated Fib-4 index, compared with those without MAFLD (**Figure 2B**).

**Table 2:** Ultrasound characteristics of patients with type 2 diabetes stratified by MAFLD status and fibrosis severity.

Variables	Patients without MAFLD (n = 85)	Patients with MAFLD and Fib-4 < 1.3 (n = 119)	Patients with MAFLD and Fib-4 ≥ 1.3 (n = 66)	p value
LA (mm)	32.94 ± 3.67	33.08 ± 3.32	33.54 ± 4.01	0.69
IVS (mm)	10.19 ± 1.48	10.06 ± 1.52	10.23 ± 1.52	0.23
LVPW (mm)	9.51 ± 0.97	9.23 ± 1.54	9.42 ± 0.96	0.24
LVEDD (mm)	44.62 ± 4.11	44.51 ± 2.97	45.58 ± 3.56	0.12
LVESD (mm)	28.11 ± 2.98	27.98 ± 2.19	28.9 ± 2.43	0.12
LVMi (g/m^2^)	93.29 ± 10.3	95.53 ± 12.88	98.15 ± 13.54	0.24
LVEF (%)	66.28 ± 4.85	66.54 ± 4.21	66.42 ± 4.89	0.84
E/e’ratio	10.75(8.66,12.29)	9.14(7.83,10.59)	11.62(10.64,13.43)	***<0.001***
LV GLS	17.69 ± 2.97	16.35 ± 3.12	15.33 ± 3.19	***<0.001***

Sample size, n = 270. Data are expressed as means± SD, medians and IQR (in parenthesis) or relative percentages. Differences between the three groups were tested by using the one-way analysis-of-variance (ANOVA) for normally distributed variables, the Kruskal-Wallis test for non-normally distributed variables (such as diabetes duration, serum triglycerides and liver enzymes) and the chi-squared test.

E/e’, peak early diastolic transmitral flow velocity to early diastolic mitral annular tissue velocity; IVS, Interventricular Septum; LVPW, Left Ventricular Posterior Wall; LVEDV, Left Ventricular end-diastolic volume; LVESV, Left Ventricular end-systolic volume; LVMI, Left Ventricular Mass Index; LVEF, Left Ventricular Ejection Fraction; LV GLS, Left Ventricular Global Longitudinal Strain; MAFLD, metabolic dysfunction-associated fatty liver disease.

Bold values indicate statistical significance (P < 0.05).

### 3.2 Ultrasound characteristics of patients dichotomized by the TyG index

Among 185 patients with MAFLD, 93 were classified into the low-TyG group and 92 into the high-TyG group. Compared with the low-TyG group, the high-TyG group had larger LVEDD (P = 0.17) and LVESD (P = 0.29), alongside impaired diastolic (E/e′: 9.14 vs. 10.75, p < 0.001) and systolic function (GLS: 15.11 ± 2.62% vs. 16.84 ± 4.85%; P<0.001) despite preserved LVEF (**Table 3**).

**Table 3:** Echocardiographic parameters of T2DM patients with MAFLD between low TyG index and high TyG index.

Variables	Low TyG index (n = 93)	High TyG index (n = 92)	p value
LA (mm)	32.94 ± 3.67	33.08 ± 3.32	0.33
IVS (mm)	10.07 ± 1.47	10.45 ± 1.36	0.38
LVPW (mm)	9.40 ± 1.06	9.30 ± 1.04	0.56
LVEDD (mm)	44.68 ± 3.93	44.82 ± 2.60	0.17
LVESD (mm)	28.07 ± 2.52	28.34 ± 2.60	0.29
LVMi (g/m^2^)	88.22 ± 2.76	91.02 ± 4.62	0.23
LVEF (%)	65.75 ± 4.79	64.33 ± 5.25	0.69
E/e’ ratio	9.14(7.83,10.59)	10.75(8.66,12.29)	***<0.001***
LV GLS	16.84 ± 4.85	15.11 ± 2.62	***<0.001***
TyG Index	8.04(7.74,8.51)	9.25(9.02,9.69)	***<0.001***

Sample size, n = 185 (patients with MAFLD). Data are expressed as means± SD, medians and IQR (in parenthesis) or relative percentages. Patients were stratified into low and high TyG groups based on the median TyG index (cutoff = 9.03). Differences between the two groups were assessed using the independent-samples t-test or the Mann–Whitney U test for continuous variables and the chi-square test for categorical variables.

E/e’, peak early diastolic transmitral flow velocity to early diastolic mitral annular tissue velocity; IVS, Interventricular Septuml; LVPW, Left Ventricular Posterior Wall; LVEDV, Left Ventricular end-diastolic volume; LVESV, Left Ventricular end-systolic volume; LVMI, Left Ventricular Mass Index; LVEF, Left Ventricular Ejection Fraction; LV GLS, Left Ventricular Global Longitudinal Strain.

Bold values indicate statistical significance (P < 0.05).

### 3.3 Associations of MAFLD severity with SLVD

**Figure 3:**
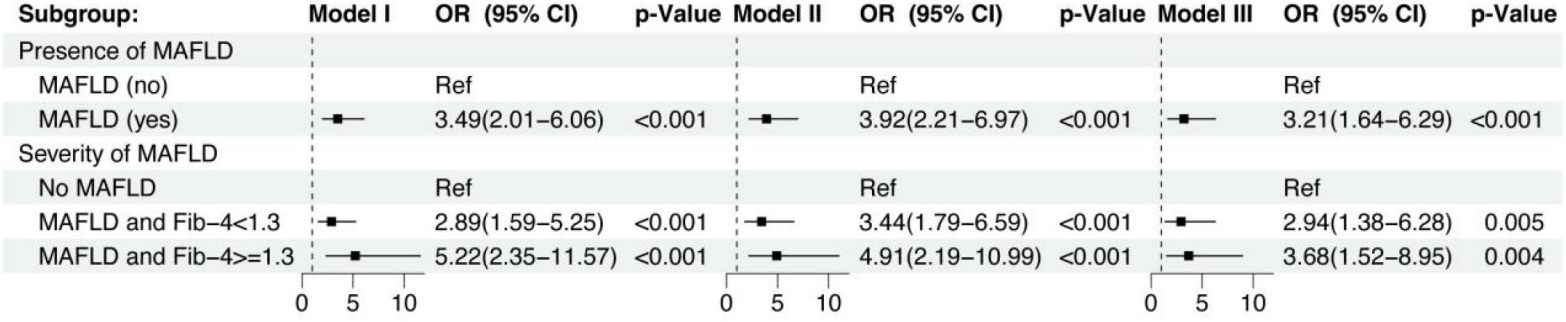
Association between MAFLD severity and the risk of SLVD in patients with T2DM. Forest plot shows odds ratios (ORs) and 95% confidence intervals (CIs) for SLVD in patients with MAFLD and Fib-4 < 1.3 and MAFLD and Fib-4 ≥ 1.3, using No MAFLD patients as the reference group. Model I: unadjusted; Model II: adjusted for age and sex; Model III: adjusted for age, sex, BMI, Plt, Diabetes duration, FPG, HbA1c, TC, HDL-C, ALT, antidiabetic medications (metformin, insulin and SGLT-2 inhibitors).

Binary logistic regression confirmed MAFLD as an independent predictor of SLVD (LV GLS<18%) (**Table 4**). The presence of MAFLD was independently associated with impaired LV GLS across all models (Model 3: adjusted OR = 3.21, 95% CI: 1.64–6.29, p=0.001). Stratified by fibrosis severity, patients with MAFLD and low fibrosis risk (Fib-4 <1.3) exhibited higher risk of impaired GLS (Model 3: OR = 2.94, 95% CI: 1.38–6.28, p = 0.005) versus non-MAFLD controls, while those with indeterminate/advanced fibrosis (Fib-4 ≥1.3) demonstrated a further elevated risk (Model 3: OR = 3.68, 95% CI: 1.52–8.95, p = 0.004) (**Figure 3**). These findings establish MAFLD and hepatic fibrosis progression as independent predictors of SLVD in T2DM, persisting after adjustment for cardiometabolic confounders. Collinearity diagnostics for the fully adjusted models are summarized in **Supplementary Table S1** and did not show evidence of problematic multicollinearity. 

**Table 4:** Association between the presence and severity of MAFLD and the risk of SLVD in patients with type 2 diabetes.

Variables	Model I		Model II		Model III	
	Odds ratio (95%CI)	p-value	Odds ratio (95%CI)	p-value	Odds ratio (95%CI)	p-value
Presence of MAFLD						
MAFLD(yes vs no)	3.49(2.01-6.06)	***<0.001***	3.92(2.21-6.97)	<0.001	3.21(1.64-6.29)	***0.001***
Severity of MAFLD						
No MAFLD (n = 85)	Ref	-	Ref	-	Ref	-
MAFLD and Fib-4<1.3(n= 119)	2.89(1.59-5.25)	***<0.001***	3.44(1.79-6.59)	***<0.001***	2.94(1.38-6.28)	***0.005***
MAFLD and Fib-4≥1.3(n= 66)	5.22(2.35-11.57)	***<0.001***	4.91(2.19-10.99)	***<0.001***	3.68(1.52-8.95)	***0.004***

Sample size, n = 270.Model I: unadjusted; Model II: adjusted for age and sex; Model III: adjusted for age, sex, BMI, Plt, DM, HbA1c, FPG, TC, HDL-C, ALT, and antidiabetic medication use (metformin, insulin and SGLT2 inhibitors). Odds ratios (ORs) and 95% confidence intervals (CIs) are reported. For the MAFLD severity analysis, the reference category is patients without MAFLD.

Bold values indicate statistical significance (P < 0.05).

### 3.4 Associations of TyG Index with SLVD

To evaluate the TyG index’s predictive value for SLVD in T2DM with MAFLD, multivariable logistic regression was performed using three hierarchical models. The TyG index demonstrated significant associations with SLVD across all models. Per 1-unit increase in TyG: Model 1 (unadjusted): OR = 2.84 (95% CI: 1.69–4.78; P < 0.001);Model 2 (age/sex-adjusted): OR = 2.82 (1.65–4.83; P < 0.001);Model 3 (fully adjusted*): OR = 2.73 (1.49–5.03; P = 0.001) (**Table 5**).

**Table 5:** Association between TyG index and the risk of SLVD in patients with type 2 diabetes and MAFLD.

Variables	Model I		Model II		Model III	
	Odds ratio (95%CI)	p-value	Odds ratio (95%CI)	p-value	Odds ratio (95%CI)	p-value
TyG index(n=185)	2.84(1.69-4.78)	***<0.001***	2.82(1.65-4.83)	***<0.001***	2.73(1.48-5.03)	***0.001***

Sample size, n = 185.Model I: unadjusted; Model II: adjusted for age and sex; Model III: adjusted for age, sex, BMI, Plt, DM, HbA1c, FPG, TC, HDL-C, ALT, and antidiabetic medication use (metformin, insulin and SGLT2 inhibitors).

Bold values indicate statistical significance (P < 0.05).

### 3.5 Combined effects of TyG index and Fib-4 index

To elucidate the combined effects of hepatic fibrosis severity and IR on SLVD in T2DM, we stratified MAFLD patients by Fib-4 index (<1.3 vs. ≥1.3) and TyG index (<9.03 vs. ≥9.03). Using the low-risk group (Fib-4<1.3 + TyG<9.03) as reference, multivariable logistic regression (Model III*) revealed: Elevated TyG alone (Fib-4<1.3 + TyG≥9.03): OR = 5.77 (95% CI: 1.97–16.70; P = 0.001). Combined advanced fibrosis + high TyG (Fib-4≥1.3 + TyG≥9.03): OR = 7.58 (95% CI: 1.72–33.40; P = 0.007).Conversely, advanced fibrosis alone (Fib-4≥1.3 + TyG<9.03) showed no significant association (P = 0.30) (**Table 6**). 

**Table 6:** Association of Fib-4 and TyG index with SLVD in patients with type 2 diabetes and MAFLD.

Variables	Model I		Model II		Model III	
	Odds ratio (95%CI)	p-value	Odds ratio (95%CI)	p-value	Odds ratio (95%CI)	p-value
Fib-4<1.3 points/TyG< 9.03 points	Ref	-	Ref	-	Ref	-
Fib-4<1.3 points/TyG > 9.03 points	6.28(2.20-17.93)	***0.001***	6.17(2.14-17.81)	***0.001***	5.77(1.97-16.70)	***0.001***
Fib-4>1.3 points/TyG < 9.03points	1.93(0.72-5.21)	0.19	2.11(0.70-6.41)	0.19	1.99(0.60-6.55)	0.30
Fib-4>1.3 points/TyG>9.03points	6.97(1.93-25.22)	***0.001***	7.45(1.90-29.43)	***0.004***	7.58(1.72-33.40)	***0.007***

Sample size, n = 185.MAFLD severity was stratified using the Fib-4 index as mild (<1.3) and advanced (≥1.3). Patients were further divided into four groups according to Fib-4 (<1.3 vs ≥1.3) and TyG (below vs above the median, 9.03), with the Fib-4 <1.3/TyG< 9.03 points group used as the reference category. Model I: unadjusted; model II: adjusted for age and sex; Model III: adjusted for age, sex, BMI, Plt, DM, HbA1c, FPG, TC, HDL-C, ALT, and antidiabetic medication use (metformin, insulin and SGLT2 inhibitors).

Bold values indicate statistical significance (P < 0.05).

### 3.6 Reproducibility of LV GLS measurements

Intra- and inter-observer reproducibility for LV GLS was excellent. The intra-observer ICC was 0.941 (95% CI 0.876–0.972), and the inter-observer ICC was 0.888 (95% CI 0.780–0.945). These results indicate high measurement stability between repeated assessments by the same observer and across different observers. Detailed reproducibility data are provided in **Supplementary Table S2**.

## 4 Discussion

In this study, we found that MAFLD, hepatic fibrosis and IR, as reflected by the TyG index, were all associated with an increased likelihood of SLVD in patients with T2DM. MAFLD remained related to impaired LV GLS after adjustment for conventional cardiometabolic risk factors, and higher TyG values were independently associated with SLVD among patients with MAFLD. Moreover, the combination of a high TyG index and advanced fibrosis was linked to the highest odds of SLVD. Although the cross-sectional design precludes causal inference, these findings are consistent with the concept that metabolic and fibrotic burden may contribute jointly to early myocardial impairment in T2DM.

First, our results support MAFLD as an independent correlate of SLVD. This is in line with previous studies showing that, even after adjustment for traditional cardiovascular risk factors, MAFLD is associated with impaired LV GLS, elevated LV filling pressure and adverse cardiac remodeling (22, 23). In the TOPCAT trial, advanced liver fibrosis was present in 37.5% of patients with HFpEF and was associated with higher hospitalization and mortality (24). Similarly, the Kailuan cohort found that moderate and severe MAFLD increased incident heart failure risk by 51% and 57% respectively (25). Respectively, So-Armah et al. reported that a Fib-4 value greater than 3.25 was associated with an increased risk of HFpEF (26). Our findings extend previous work by linking how severe liver fibrosis is to LV GLS, and by indicating that even patients with low Fib-4 values (Fib-4 <1.3) can already show mild LV systolic impairment. Overall, our findings indicate that liver fibrosis is associated with early myocardial impairment, while LV GLS serves as a sensitive echocardiographic marker for detecting these early changes.

Second, we found that the TyG index was independently associated with SLVD in T2DM patients with MAFLD. This extends prior observations that higher TyG levels are associated with lower LV GLS in patients with coronary heart disease and chronic heart failure (27, 28), and it corroborates evidence linking elevated TyG index to atherosclerotic CVD (29) and heart failure incidence (30). In our cohort, each 1-unit increase in TyG was associated with an approximately three-fold higher odds of impaired LV GLS, which is numerically greater than the 1.52-fold increase reported in obese populations (31). This difference may reflect the greater IR burden and hepatic steatosis in our diabetic cohort, although differences in study design and population also need to be considered. From a mechanistic perspective, sustained IR and atherogenic dyslipidemia may promote lipotoxicity, mitochondrial dysfunction and oxidative stress, leading to impaired myocardial energetics and extracellular matrix remodeling (32–34). Taken together, these data support the TyG index as a simple clinical marker associated with occult myocardial dysfunction in T2DM, while recognizing that the underlying biology remains to be fully elucidated.

Beyond the TyG index, other simple lipid-derived markers have been proposed as proxies of IR and CVD. In particular, the triglycerides-to-HDL cholesterol ratio (TG/HDL) has gained increasing attention because it reflects both an atherogenic lipid profile and impaired insulin sensitivity (35). Recent data in individuals with prediabetes showed that TG/HDL, but not the TyG index, was independently associated with arterial stiffness, a widely used marker of subclinical vascular damage and future cardiovascular events (36). In another high-risk setting, patients with familial hypercholesterolemia and subclinical atherosclerosis exhibited a more adverse metabolic profile, including higher TyG and TG/HDL values, compared with those without subclinical atherosclerosis (37). Taken together, these findings suggest that TG/HDL and TyG may capture partially distinct aspects of the cardiometabolic continuum—TG/HDL being more closely related to an atherogenic dyslipidemia pattern and vascular stiffness, whereas TyG appears to be more directly linked to IR in glucose-metabolic disorders.

Third, we observed that the coexistence of high TyG and advanced fibrosis was associated with markedly higher odds of SLVD than either factor alone. Although advanced hepatic fibrosis was not significantly associated with SLVD in isolation in our cohort, previous studies have shown that histologically confirmed NASH and severe liver fibrosis are linked to altered cardiac structure, impaired diastolic function, increased left atrial volume and greater LV mass, all indicative of early myocardial dysfunction (38). In our study, the subgroup with both high TyG and high Fib-4 had an odds ratio of 7.58 for SLVD, suggesting that severe hepatic fibrosis may adversely affect myocardial mechanics and that this effect is amplified in the presence of systemic IR. At a mechanistic level, the coexistence of hepatic fibrosis and IR may create a milieu of hemodynamic stress, metabolic derangement and neurohormonal activation that adversely affects myocardial structure and function. In chronic liver disease, systemic inflammation, altered gut permeability and activation of the renin-angiotensin-aldosterone system, further enhanced by IR related lipotoxicity and oxidative stress (39, 40), provide a plausible mechanistic basis for this pattern. However, given the limited subgroup sample size and the observational design, these findings should be interpreted with caution.

From a clinical standpoint, our results suggest that simple, routinely available markers such as ultrasound-based MAFLD assessment, the Fib-4 index, and the TyG index may help identify T2DM patients at higher risk of early cardiac injury. In particular, patients with MAFLD and concomitantly elevated Fib-4 and TyG values may benefit from closer cardiovascular surveillance and optimization of cardioprotective therapies. Agents such as SGLT2 inhibitors have been shown to reduce heart failure hospitalization and cardiovascular death across the spectrum of LVEF and improve several cardiometabolic parameters (41, 42). However, as our study was not designed to evaluate treatment effects or establish therapeutic thresholds, the TyG/Fib-4 combinations identified should be viewed as exploratory markers for risk stratification rather than prescriptive cut-offs for clinical decision-making. Prospective studies are needed to determine whether integrating liver-related and IR-related indices into routine risk assessment improves outcomes beyond standard care.

Nevertheless, several limitations must be acknowledged. First, the cross-sectional design prevents causal inference and precludes evaluation of progression from subclinical dysfunction to overt heart failure. Second, this was a single-center study from a tertiary hospital, which may introduce selection bias and limit generalizability; validation in larger, multicenter and multiethnic cohorts is required. Third, liver fibrosis was assessed by Fib-4 index rather than liver biopsy or elastography; although Fib-4 is endorsed for risk stratification, it shows age-related bias with reduced specificity in patients aged ≤35 or ≥65 years (43). We used a uniform cut-off of 1.3 without age-stratified thresholds, which may have led to misclassification of fibrosis risk in the youngest and oldest participants. Fourth, we did not have complete information on statin and antihypertensive therapy, so residual confounding by these treatments cannot be excluded. Fifth, although TG and HDL were measured in our cohort, we did not pre-specify or calculate the TG/HDL ratio as an exposure variable, and therefore we could not explore whether TG/HDL provides complementary information to the TyG index for SLVD, which should be examined in future studies. Sixth, echocardiographic assessment focused on LV GLS, and we did not systematically evaluate right ventricular function or exercise capacity. Finally, unmeasured confounders including physical activity, nutritional status, hormone replacement therapy and inflammatory markers may have influenced the associations.

Looking forward, longitudinal studies are essential to confirm the temporal sequence of the metabolism-liver-heart axis and to determine whether TyG/Fib-4 thresholds predict progression to heart failure. Future studies should also incorporate both TyG and TG/HDL in the same cohorts to directly compare their relative and combined value for predicting SLVD in T2DM patients with MAFLD. Large biobank cohorts and multiethnic registries should be leveraged to validate our proposed clinical algorithm and to refine age specific cut offs using artificial intelligence models. Mechanistic studies are needed to delineate how IR and hepatic fibrosis interact at the molecular level to impair myocardial energetics, for example through shared pathways involving oxidative stress, mitochondrial dysfunction and gut dysbiosis. Randomized trials are needed to test whether initiating SGLT-2 inhibitors, GLP-1 receptor agonists or emerging liver directed agents in high risk patients improves LV GLS and prevents clinical heart failure. Age adjusted biomarkers such as enhanced liver fibrosis (ELF) or imaging modalities including transient elastography and proton density fat fraction Magnetic Resonance Imaging (MRI) should be considered for future studies to overcome the limitations of Fib-4. Ultimately, integrating hepatic and cardiac assessment into routine diabetic care and adopting a multidisciplinary model will enable precision prevention of diabetic cardiomyopathy.

## 5 Conclusion

MAFLD and its fibrotic progression are closely associated with SLVD in T2DM, and the TyG index further amplifies this risk. Combined assessment of Fib-4 index and TyG index provides a simple, non-invasive stratification strategy that may facilitate the early identification of high-risk individuals with preserved LVEF, which may help guide timely preventive interventions.

## Data Availability

The raw data supporting the conclusions of this article will be made available by the authors, without undue reservation.
