# Supplementary material for: TyG index and MAFLD severity are associated with subclinical LV dysfunction in T2DM patients: a cross−sectional study
Source: Front Endocrinol (Lausanne). 2026 Jan 27;16:1749989. doi: 10.3389/fendo.2025.1749989 (PMC12886020; doi:10.3389/fendo.2025.1749989)
Supplement: Supplementary file 1 [file Table1.docx]

**Supplementary Table S1. Variance inflation factors (VIFs) for predictors included in the fully adjusted model (Model III).**

|  | VIF | Tolerance |
| --- | --- | --- |
| TyG index | 1.725 | 0.580 |
| age | 1.310 | 0.763 |
| sex | 1.258 | 0.795 |
| BMI | 1.266 | 0.790 |
| DM | 1.206 | 0.829 |
| HbA1c | 1.075 | 0.931 |
| FPG | 1.758 | 0.569 |
| TC | 2.243 | 0.446 |
| TG | 2.113 | 0.473 |
| HDL-C | 1.757 | 0.569 |
| ALT | 1.041 | 0.961 |
| metformin | 1.059 | 0.944 |
| insulin | 1.164 | 0.859 |
| SGLT-2 | 1.073 | 0.932 |

VIF values >5 were considered indicative of problematic multicollinearity. All VIFs in this model ranged from 1.04 to 2.24, indicating no evidence of problematic multicollinearity.

**Supplementary Table S2. Intra-observer and inter-observer variability.**

|  | **Intra-observer** | |  | **Inter-observer** | |
| --- | --- | --- | --- | --- | --- |
|  | **ICC** | **95% CI** |  | **ICC** | **95% CI** |
| LV GLS | 0.941 | 0.876-0.972 |  | 0.888 | 0.780-0.945 |
